# Gas Chromatography–Mass Spectrometry Detection of Thymoquinone in Oil and Serum for Clinical Pharmacokinetic Studies

**DOI:** 10.3390/ijms242216431

**Published:** 2023-11-17

**Authors:** A. Tekbaş, S. Bremer-Streck, D. K. Wissenbach, F. T. Peters, M. von Lilienfeld-Toal, Z. Soonawalla, F. Rauchfuß, U. Settmacher, U. Dahmen

**Affiliations:** 1 Department of General, Visceral and Vascular Surgery, Jena University Hospital, Friedrich Schiller University Jena, Am Klinikum 1, 07747 Jena, Germany; 2 Research Programme “Clinician Scientist Programme”, Interdisciplinary Center of Clinical Research, Medical Faculty Jena, Jena University Hospital, Friedrich Schiller University Jena, Salvador-Allende-Platz 29, 07747 Jena, Germany; 3 Experimental Transplantation Surgery, Jena University Hospital, Friedrich Schiller University Jena, Am Klinikum 1, 07747 Jena, Germany; 4 Institute of Clinical Chemistry and Laboratory Diagnostics, Centralised Diagnostic Laboratory Services, Jena University Hospital, Friedrich Schiller University Jena, Am Klinikum 1, 07747 Jena, Germany; 5 Institute for Forensic Medicine, Jena University Hospital, Friedrich Schiller University Jena, Am Klinikum 1, 07747 Jena, Germany; 6 Institute for Diversity Medicine, Ruhr-University Bochum, Universitaetsstr. 105, 44789 Bochum, Germany; 7 Hepato-Pancreato-Biliary Surgery, Oxford University Hospitals NHS Foundation Trust, Headley Way, Headington, Oxford OX3 9DU, UK

**Keywords:** GC-MS, *Nigella sativa*, pharmacokinetics, therapeutic use, thymoquinone

## Abstract

Thymoquinone (TQ) is the primary component of *Nigella sativa* L. (NS) oil, which is renowned for its potent hepatoprotective effects attributed to its antioxidant, anti-fibrotic, anti-inflammatory, anti-carcinogenic, and both anti- and pro-apoptotic properties. The aim of this work was to establish a method of measuring TQ in serum in order to investigate the pharmacokinetics of TQ prior to a targeted therapeutic application. In the first step, a gas chromatography–mass spectrometry method for the detection and quantification of TQ in an oily matrix was established and validated according to European Medicines Agency (EMA) criteria. For the assessment of the clinical application, TQ concentrations in 19 oil preparations were determined. Second, two serum samples were spiked with TQ to determine the TQ concentration after deproteinization using toluene. Third, one healthy volunteer ingested 1 g and another one 3 g of a highly concentrated NS oil 30 and 60 min prior to blood sampling for the determination of serum TQ level. After the successful establishment and validation of the measurement method, the highest concentration of TQ (36.56 g/L) was found for a bottled NS oil product (No. 1). Since a capsule is more suitable for oral administration, the product with the third highest TQ concentration (No. 3: 24.39 g/L) was used for all further tests. In the serum samples spiked with TQ, the TQ concentration was reliably detectable in a range between 5 and 10 µg/mL. After oral intake of NS oil (No. 3), however, TQ and/or its derivatives were not detectable in human serum. This discrepancy in detecting TQ after spiking serum or following oral ingestion may be attributed to the instability of TQ in biomatrices as well as its strong protein binding properties. A pharmacokinetics study was therefore not viable. Studies on isotopically labeled TQ in an animal model are necessary to study the pharmacokinetics of TQ using alternative modalities.

## 1. Introduction

*Nigella sativa* L. (NS) belongs to a family of medicinal plants that are safe alternatives to allopathic drugs with fewer side effects [1,2]. As a part of an overall holistic approach to health, it has long been used for traditional purposes in the Middle East, being native to Southern Europe, North Africa and Southwest Asia [3,4]. The essential oil of NS is obtained from the seeds through cold pressing, and thymoquinone (TQ, IUPAC name: 2-methyl-5-(propan-2-yl)cyclohexa-2,5-diene-1,4-dione) constitutes up to 50% of the main active components in the oil [2,5]. In our systematic review published on this topic, we pointed out that TQ has a wide therapeutic window with strong antioxidant, anti-inflammatory, anti-fibrotic, anti-/proapoptotic and anti-carcinogenic effects [4]. This characterizes TQ as a promising drug candidate for various inflammatory and neoplastic diseases. There have been many studies published on the pharmacology of TQ, but there is sparse information regarding its pharmacokinetics [6]. However, for proper use in a clinical study, the determination of pharmacokinetics is essential.

Hence, our aim was to develop a method and evaluate the TQ content in commercially available NS oil preparations through gas chromatography–mass spectrometry (GC-MS). Subsequently, we sought to assess the method’s applicability to a protein-rich aqueous matrix like human serum, as a crucial step for conducting pharmacokinetic studies involving healthy volunteers.

## 2. Results

### 2.1. Method Development and Validation Results

**Figure 1:**
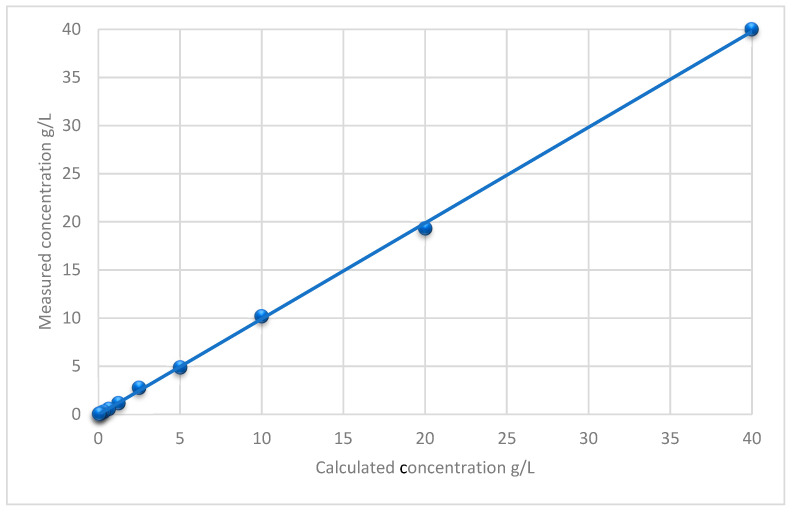
Linearity curve for concentrations between 0.03 g/L and 40 g/L with regression line.

**Figure 2:**
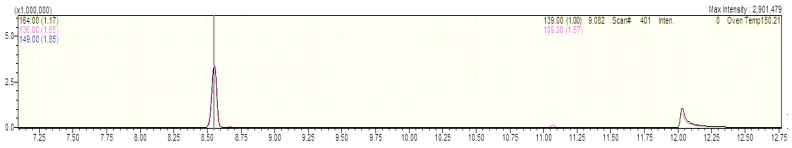
Method validation: GC-MS, extracted ion chromatogram, with the peak for TQ detected in NS oil No. 3 after 8.55 min and IS-M after 12.05 min.

Linearity was established between 0.03 and 40 g/L. The linear regression coefficient was r = 0.99 (Figure 1). The intra-assay precision was 7.3% (relative standard deviation, RSD). For the inter-assay precision, 7.2% (RSD) was calculated for 4.8 g/L and 7.6% (RSD) for 24.4 g/L. The lower limit of quantification (LLOQ) was 0.03 g/L (RSD 4%). The concentration of TQ remained stable under the mentioned storing conditions (Table 1). The RSD of the slopes in the addition curves after spiking six different oil samples with defined TQ concentrations was 6.6%. The accuracy of recovery was 0.5 to 7.2%. TQ showed a carry-over of 0.001 g/L. This corresponds to 3.3% of the LLOQ. The accuracy value for dilution integrity at 2-fold dilution was 4.6%. Figure 2 shows the extracted ion chromatogram with the peak for TQ detected in NS oil No. 3 and IS-M (internal standard for method evaluation).

### 2.2. Quantification of TQ in Different NS Oil Products

With the validated method, 19 different oil products were analyzed for their TQ concentration (No. 1–19, Table 2). The highest concentration was found in oil No. 17 (c = 36.56 g/L), followed by No. 19 (c = 27.92 g/L) and No. 3 (c = 24.39 g/L). All three products were produced from Ethiopian NS seeds. Oil No. 3 is a capsulated product and was therefore chosen for the test with human samples from healthy volunteers.

### 2.3. Quantification of TQ in Serum Samples and Detection of TQ Metabolites in Urine: Preliminary Results

As TQ quantification should be applied for pharmacokinetic studies, preliminary results for TQ in biomatrices were obtained.

**Figure 3:**
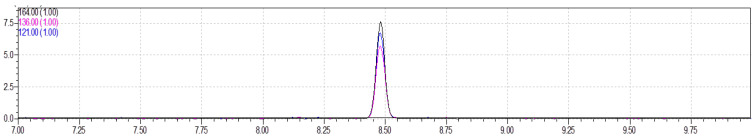
Serum spiked with 5 µg/mL TQ. GC-MS, extracted ion chromatogram, with the peak for TQ after 8.48 min.

**Figure 4:**
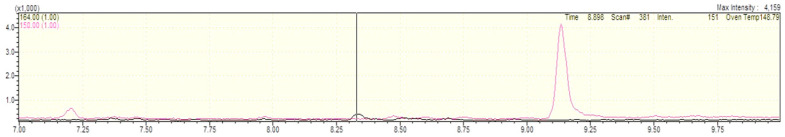
GC-MS, extracted ion chromatogram: TQ was not detected in serum after oral application (two capsules of NS oil No. 3). Peak indicates IS-S (internal standard for serum analyses) after 9.14 min.

Therefore, TQ was spiked in human serum (at concentrations of 5 µg/mL and 10 µg/mL) and successfully detected via GC-MS (Figure 3). However, TQ was not detected in authentic human serum samples after oral intake of two (Figure 4, normal dose) and six No. 3 capsules (high dose), corresponding to 0.024 g and 0.072 g TQ per person.

In addition, TQ and/or its metabolites were not detected in urine via untargeted GC-MS and LC-MS/MS analysis [7,8] after application of a high-dose TQ. Furthermore, TQ showed significant matrix-dependent decay up to 80% within 240 min after spiking whole blood with 10 mg/mL TQ and within 30 min in serum.

## 3. Discussion

In this study, we developed and validated a GC-MS-based method to measure the concentration of TQ accurately and precisely in an oily matrix. With this method, we determined the TQ concentration in 19 oil products in order to select one with a high TQ concentration for further clinical pharmacokinetic studies in humans.

High-performance liquid chromatography (HPLC), high-performance thin-layer chromatography, differential pulse polarographic and GC-MS methods have been reported for TQ quantification in black seed oil [9,10,11,12,13]. It is desirable to optimize these methods for the detection of TQ from blood/serum in order to define its pharmacokinetic profile and to assess its properties in a preclinical setting. However, due to their high reactivity as fast redox cycling compounds as well as their easy adduction to electron-rich nucleophiles, the analysis of quinones is challenging [14,15].

Interestingly, we were able to detect and quantify TQ in spiked serum. However, TQ was not detected in authentic serum samples, even after the application of higher doses.

One reason for this might be the observed instability of TQ in biomatrices, as was also described by Alkharfy et al. [16]. In rabbits treated with 5 mg/kg TQ iv, the plasma concentration versus time curve showed a bi-exponential decline process [16]. Another reason is probably low bioavailability due to the high affinity of TQ for proteins, as described/confirmed by El-Najjar et al. [6]. In this study, the average recovery of TQ from serum was 2.5% at 10 µg/mL TQ and 72% at 100 µg/mL [6]. The authors stated that HPLC does not appear suitable for pharmacokinetic studies at low TQ concentrations because the extent of protein binding is high, and thus the concentration of free TQ might be below the detection limit [6].

Based on the results of El-Najjar et al., TQ may bind covalently and non-covalently to serum components, limiting the use of conventional analytical methods for its detection and quantification in plasma and the analysis of its bioavailability [6].

Alkharfy et al. [16] developed an HPLC assay to determine low concentrations of TQ in rabbit plasma. After intravenous (iv) application of TQ at a dose of 5 mg/kg in rabbits, quantification of TQ was possible. The limit of quantification was indicated as 0.408 µg/mL. The elimination half-life (T_1/2_) of TQ was 99.71 ± 22.41 min based on a two-compartment pharmacokinetic model. In a consecutive work, Alkharfy et al. tested the iv and oral bioavailability of TQ, also in rabbits [17]. Following an iv dose of 5 mg/kg, the T_1/2_ was similar to that reported before (89.69 ± 12.82 min in a two-compartment model). The oral administration showed a slower absorption characteristic at a dose of 20 mg/kg (T_1/2_ 225.61 ± 9.08 min, one-compartment model). All in all, TQ was quickly eliminated from the plasma. The bioavailability of TQ was estimated as 58%. Similar to El-Najjar et al., Alkharfy et al. specified the percentage of TQ protein binding in rabbit and human plasma to be 99.19 ± 0.29 and 98.99 ± 0.32, respectively. Iqbal et al. [18] presented similar results in layer chickens. In their experiments, the limit of quantification was even indicated as 0.05 µg/mL. The elimination half-life after the application of 5 mg/kg TQ iv (non-compartmental pharmacokinetic) was 0.978 ± 0.205 h.

Considering these studies however, it is essential to state that the non-detectability of TQ in our in vivo study might also have been due to the very low dosage we used compared to the described studies. The relative dose of ingested TQ per volunteer (70 kg) in our study was 0.3 mg/kg for the normal dose (0.024 g, dosing per intake) and 1 mg/kg for the high dose (0.072 g, recommended dose of two capsules three times a day). This is a very low fraction of the dosages used in the cited studies and seems to be insufficient considering the complex pharmacokinetic behavior of TQ in plasma described by El-Najjar et al. [6].

Moreover, intravenous administration of TQ circumvents the metabolic processes, rendering it more susceptible to detection in contrast to the oral route with subsequent metabolism. Additionally, the challenge of suboptimal absorption from the gastrointestinal tract into systemic circulation may hinder its efficacy when administered orally. A noteworthy publication authored by Ansar et al. proposes an innovative approach involving the formulation of TQ with nanostructured lipid carriers to enhance the bioavailability of oral preparations [19]. This strategic advance appears to offer a promising solution to the challenges associated with the oral route of TQ administration.

Even though metabolites such as glutathione conjugates have been described in the literature [20], corresponding TQ metabolites could not be detected in the preliminary experiments via untargeted GC-MS in serum and untargeted LC-MS/MS in urine. These results strengthen the hypothesis that the detection of TQ after oral administration is complicated by compound instability in biomatrices and/or additional unknown metabolic processes.

Furthermore, the limit of quantification we achieved during our method validation process was much higher than the one achieved by Alkharfy et al. in plasma [16]. The difference in the values is probably due to the fact that the measurements were performed in different matrices (oil and plasma, respectively) and with different methods.

Following these results, subsequent investigations involve studies utilizing isotopically labeled TQ in an animal model to explore TQ pharmacokinetics in vivo, employing alternative modalities such as computed tomography.

## 4. Materials and Methods

### 4.1. Experimental Design

The protocol for the experimental design was adopted from Johnson-Ajinwo et al., 2014 [9] and Alkharfy et al., 2013 [16]. First, GC-MS was established and validated for the detection of TQ in an oily matrix according to European Medicines Agency (EMA) criteria. With this method, the TQ concentration (c) in 19 commercially available and randomly chosen Egyptian and Ethiopian oil preparations was quantified. Then, after measuring TQ concentrations in spiked serum samples, the determination of TQ was carried out in two healthy volunteers. One was treated with 1 g and the other with 3 g of a highly concentrated NS oil—according to the measurement results—30 and 60 min prior to blood sampling.

### 4.2. Chemicals and Reagents

TQ, thymol (TM) and toluene (T) were purchased from Sigma Aldrich (Steinheim, Germany). N-hexane and chloroform for gas chromatography and 4-nitrophenol were obtained from Merck (Darmstadt, Germany). Gradient-grade methanol was purchased from Carl Roth (Karlsruhe, Germany).

In order to improve analytical robustness, quantitative results were obtained after calibration using an internal standard (IS). Therefore, for method evaluation, 25 mg of 4-nitrophenol was dissolved in 2.5 mL of chloroform and diluted with 200 mL of n-hexane (IS-M). For TQ quantification in human serum, TM diluted with T in a concentration of 0.1 mg/L was proven to be suitable as IS (IS-S). All reagents used in the experiments were of analytical grade.

### 4.3. Human Serum Samples

Human serum–gel samples (collection tubes with a gel separator) were obtained from two native healthy volunteers 30 and 60 minutes (min) after oral intake of NS seed oil capsules (No. 3). For the serum samples spiked with 5 mg/L and 10 mg/L TQ, a dilution was prepared using the stock solution (see Section 4.4.2) and serum. Serum was separated via centrifugation at 2500× *g* for 10 min. Written informed consent was obtained from both participants. The study was approved by the local ethics committee (No. 2020-2033-BO).

### 4.4. Calibration Standards and Quality Control Samples

#### 4.4.1. Method Validation

A standard stock solution for TQ in n-hexane was prepared at a concentration of 40 g/L. This solution was stored at −4 °C for a maximum of five weeks.

For the calibration solution, a mixture was prepared by vortexing 50 µL of linseed oil, 50 µL of the TQ stock solution (40 g/L), and 900 µL of n-hexane in a labeled Eppendorf reaction vessel. Subsequently, 100 µL of this mixture was vortexed with 900 µL of the internal standard (IS-M).

The preparation of the quality control samples using two oil samples (oil No. 2 and No. 3) was conducted in accordance with the description provided in Section 4.6.3.

#### 4.4.2. Clinical Application

A standard TQ stock solution in n-hexane was prepared at a concentration of 100 mg/L TQ and stored at −4 °C for a maximum of five weeks.

The calibration solution was prepared by diluting the stock solution with serum to achieve a concentration of 10 mg/L of TQ. Deproteinization was carried out using further dilution with IS-S (1:2). All working solutions were freshly prepared for each day of experimentation.

### 4.5. Sample Preparation

#### 4.5.1. Method Validation

A total of 50 µL of NS oil (sample No. 1–19) was added to 950 µL of n-hexane in a labeled Eppendorf reaction vessel to be vortexed thoroughly. Then, 100 µL of this mixture and 900 µL of IS-M were vortexed in a labeled Eppendorf reaction vessel. Finally, 1 µL of this mixture was injected onto the GC-MS column.

#### 4.5.2. Clinical Application

Serum samples were deproteinized by adding 100 µL of the IS-S containing the deproteinization solution T (1:2). Samples were vortexed for one minute and the tubes were then centrifuged at 11,000× *g* for 5 min at room temperature (RT). Approximately 100 µL of the supernatant was transferred to 2 mL screw-top glass vials with 200 µL glass inserts and silicon septa caps (Agilent, Santa Clara, CA, USA), and 5 µL was injected for analysis.

### 4.6. GC-MS Analysis

GC-MS analysis was conducted using a Shimadzu QP 2010 GC-MS system (Shimadzu, Kyoto, Japan) equipped with a Zebron capillary column (30 m × 0.32 mm × 1 µm; Phenomenex, Torrance, CA, USA). Helium (>99.99%) with a linear velocity of 41.7 cm/s was employed as the carrier gas. The injector was configured in the split injection mode (ratio 1:5) and maintained at a temperature of 280 °C, while the column flow rate was set at 1.27 mL/min. Chromatographic separation was successfully achieved within 25.4 min. The ion source was held at a temperature of 200 °C, and ionization was carried out in the electron impact mode with an ionization energy of 70 eV. Detection was performed in the selected ion monitoring (SIM) mode. The injection volume was standardized at 1 µL. TQ and IS were identified via reference spectra matching. Quantification of TQ, with a retention time of 8.5 min, was achieved by monitoring 164 *m*/*z*, while IS, with a retention time of 12 min, was monitored at 139 *m*/*z*. Analyte/IS peak area ratios were employed for internal calibration.

For method evaluation, single-point calibration was applied using TQ at a concentration of 40 mg/mL.

#### 4.6.1. Method Validation

The method validation was performed according to the current European Medicines Agency (EMA) guidelines [21]. The validation of the developed method included linearity, precision, lower limit of quantification (LLOQ), stability, matrix effect, carry-over and dilution integrity.

#### 4.6.2. Linearity

Linearity was assessed via 2-fold measuring of the highest calibration standard (40 g/L) and ten different dilutions (20–10–5–2.5–1.25–0.625–0.3125–0.15625–0.078125–0.0390625 g/L) within ranges of the LLOQ. Sample preparation was conducted at RT (25 ± 1 °C).

#### 4.6.3. Precision

The precision of the method was repeatedly determined using two concentrations of quality control samples (4.8 g/L and 24.4 g/L), which were processed freshly on each day of experimentation. For intra-assay precision, one concentration (24.4 g/L) was measured 5 times within a run, and for inter-assay precision, three single series per day were analyzed on two different days for both concentrations. The relative standard deviation (RSD) for the precision should not exceed 15% (20% for LLOQ).

#### 4.6.4. LLOQ

The LLOQ was defined as the lowest concentration of TQ in an oil sample (precision of ≤20% (RSD)).

#### 4.6.5. Stability

Analyses of the stability of three samples of working solution were performed. The stability of the samples was tested at RT (25 ± 1 °C), after freezing (−20 °C) and after storage in the refrigerator (4–8 °C). The concentrations of analytes in solutions stored for 15 and 30 days in Eppendorf tubes were compared with the concentration in fresh samples.

#### 4.6.6. Matrix Effect

The matrix effect was determined by spiking six different TQ-free oil samples with three different concentrations (5, 10 and 20 g/L) of the compound. The RSD of the slopes in the addition curves must not exceed 15%.

#### 4.6.7. Carry-Over

The carry-over was measured by injecting a blank sample after the highest calibration standard (40 g/L). It should be less than 20% of the LLOQ for the analyte.

#### 4.6.8. Dilution Integrity

The dilution integrity was tested to quantify concentrations greater than the calibration interval. A sample was diluted 2-fold with a blank matrix (TQ-free linseed oil). The accuracy should be within ≤15%.

## 5. Conclusions

Based on our research, no previous effort has been made to quantify TQ in human serum following the oral administration of NS oil. Although a clinical pharmacokinetics study was not viable, our findings enhance the overall comprehension of TQ in a clinical context.

## Figures and Tables

**Table 1:** Stability of NS oil No. 3 in the refrigerator (4–8 °C) and after freezing (−20 °C): Demonstration of the deviation from the room temperature (RT) under storing conditions in percent (%).

Storage Duration	Storage Condition	Deviation from RT in%
15 days	4–8 °C	1.3
	−20 °C	3
30 days	4–8 °C	0.4
	−20 °C	1.6

**Table 2:** Mean TQ concentrations in g/L and relative standard deviation (RSD) in % in 19 different *Nigella sativa* (NS) oil products.

NS Oil Product	Mean TQ in g/L	RSD in%
No. 1	1.0	23.9
No. 2	4.8	0.4
No. 3	24.4	8.9
No. 4	1.15	3.4
No. 5	21.1	4.7
No. 6	0.9	5.6
No. 7	4.8	5.1
No. 8	1.3	1.9
No. 9	1.0	2.8
No. 10	2.5	2.1
No. 11	2.7	14.6
No. 12	0.6	13.3
No. 13	0.6	3.1
No. 14	1.3	9.1
No. 15	6.7	15.4
No. 16	1.0	2.2
No. 17	36.6	9.3
No. 18	3.5	5.6
No. 19	27.9	0.4

## Data Availability

The datasets analyzed during the current study are available on request from the corresponding author.

## References

[1] Bent S. (2008). Herbal medicine in the United States: Review of efficacy, safety, and regulation: Grand rounds at University of California, San Francisco Medical Center. J. Gen. Intern. Med..

[2] Ahmad A., Husain A., Mujeeb M., Khan S.A., Najmi A.K., Siddique N.A., Damanhouri Z.A., Anwar F. (2013). A review on therapeutic potential of *Nigella sativa*: A miracle herb. Asian Pac. J. Trop. Biomed..

[3] Khare C. (2004). Encyclopedia of Indian Medicinal Plants.

[4] Tekbas A., Huebner J., Settmacher U., Dahmen U. (2018). Plants and Surgery: The Protective Effects of Thymoquinone on Hepatic Injury-A Systematic Review of In Vivo Studies. Int. J. Mol. Sci..

[5] Mollazadeh H., Hosseinzadeh H. (2014). The protective effect of *Nigella sativa* against liver injury: A review. Iran. J. Basic Med. Sci..

[6] El-Najjar N., Ketola R.A., Nissila T., Mauriala T., Antopolsky M., Janis J., Gali-Muhtasib H., Urtti A., Vuorela H. (2011). Impact of protein binding on the analytical detectability and anticancer activity of thymoquinone. J. Chem. Biol..

[7] Maurer H.H., Pfleger K., Weber A.A. (2016). Mass Spectral and GC Data of Drugs, Poisons, Pesticides, Pollutants, and Their Metabolites.

[8] Maurer H.H., Wissenbach D.K., Weber A.A. (2019). Maurer/Wissenbach/Weber LC-MSn Library of Drugs, Poisons, and Their Metabolites.

[9] Johnson-Ajinwo O.R., Li W.W. (2014). Stable isotope dilution gas chromatography-mass spectrometry for quantification of thymoquinone in black cumin seed oil. J. Agric. Food Chem..

[10] Botnick I., Xue W., Bar E., Ibdah M., Schwartz A., Joel D.M., Lev E., Fait A., Lewinsohn E. (2012). Distribution of primary and specialized metabolites in *Nigella sativa* seeds, a spice with vast traditional and historical uses. Molecules.

[11] Ghosheh O.A., Houdi A.A., Crooks P.A. (1999). High performance liquid chromatographic analysis of the pharmacologically active quinones and related compounds in the oil of the black seed (*Nigella sativa* L.). J. Pharm. Biomed. Anal..

[12] Michelitsch A., Rittmannsberger A. (2003). A simple differential pulse polarographic method for the determination of thymoquinone in black seed oil. Phytochem. Anal..

[13] Velho-Pereira R.M., Barhate C.R., Kulkarni S.R., Jagtap A.G. (2011). Validated high-performance thin-layer chromatographic method for the quantification of thymoquinone in *Nigella sativa* extracts and formulations. Phytochem. Anal..

[14] Land E.J., Ramsden C.A., Riley P.A. (2004). Quinone chemistry and melanogenesis. Methods Enzymol..

[15] Li W.W., Heinze J., Haehnel W. (2005). Site-specific binding of quinones to proteins through thiol addition and addition-elimination reactions. J. Am. Chem. Soc..

[16] Alkharfy K.M., Ahmad A., Khan R.M.A., Al-Asmari M. (2013). High-performance liquid chromatography of thymoquinone in rabbit plasma and its application to pharmacokinetics. J. Liq. Chromatogr. Relat. Technol..

[17] Alkharfy K.M., Ahmad A., Khan R.M., Al-Shagha W.M. (2015). Pharmacokinetic plasma behaviors of intravenous and oral bioavailability of thymoquinone in a rabbit model. Eur. J. Drug Metab. Pharmacokinet..

[18] Iqbal S., Javeed A., Sattar A., Tanvir R. (2019). Pharmacokinetics of thymoquinone in layer chickens following oral and intravenous administration. J. Vet. Pharmacol. Ther..

[19] Zakarial Ansar F.H., Latifah S.Y., Wan Kamal W.H.B., Khong K.C., Ng Y., Foong J.N., Gopalsamy B., Ng W.K., How C.W., Ong Y.S. (2020). Pharmacokinetics and Biodistribution of Thymoquinone-loaded Nanostructured Lipid Carrier After Oral and Intravenous Administration into Rats. Int. J. Nanomed..

[20] Khalife K.H., Lupidi G. (2007). Nonenzymatic reduction of thymoquinone in physiological conditions. Free Radic. Res..

[21] European Medicines Agency (EMA) (2012). Guideline on Bioanalytical Method Validation. https://www.ema.europa.eu/en/documents/scientific-guideline/guideline-bioanalytical-method-validation_en.pdf.

